# Evaluation of the physical and antifungal effects of chlorhexidine diacetate incorporated into polymethyl methacrylate

**DOI:** 10.1590/1678-7757-2019-0039

**Published:** 2019-12-17

**Authors:** Caroline Vieira Maluf, Luciana Vieira Peroni, Lívia Rodrigues Menezes, Wagner Coutinho, Eduardo José Veras Lourenço, Daniel de Moraes Telles

**Affiliations:** 1 Universidade do Estado do Rio de Janeiro, Departamento de Prótese Dentária, Rio de Janeiro, Rio de Janeiro, Brasil.; 2 Universidade Federal do Rio de Janeiro, Instituto de Macromoléculas Eloisa Mano, Rio de Janeiro, Rio de Janeiro, Brasil.; 3 Universidade do Estado do Rio de Janeiro, Rio de Janeiro, Rio de Janeiro, Brasil.

**Keywords:** Acrylic resins, Chlorhexidine, Candida albicans, Antifungal agents, Physical properties

## Abstract

This study aimed to evaluate the physical properties and antifungal activities of polymethyl methacrylate (PMMA) acrylic resins after the incorporation of chlorhexidine diacetate salt (CDA).

**Methodology:**

First, acrylic resin specimens were fabricated with Vipi Cor^®^ and DuraLay^®^ resins with and without the incorporation of 0.5%, 1.0% or 2.0% CDA. The residual monomer and CDA release were measured at intervals ranging from 2 hours to 28 days using ultraviolet spectrometry combined with high-performance liquid chromatography. The antifungal activity against *C. albicans* was evaluated with the agar diffusion method. Fourier transform infrared spectroscopy was used to analyze the degree of resin conversion. Finally, the water sorption values of the resins were also measured.

**Results:**

The incorporated CDA concentration significantly changed the rate of CDA release (p<0.0001); however, the brand of the material appeared to have no significant influence on drug release. Subsequently, the inhibition zones were compared between the tested groups and within the same brand, and only the comparisons between the CDA 2% and CDA 1% groups and between the CDA 1% and CDA 0.5% groups failed to yield significant differences. Regarding the degrees of conversion, the differences were not significant and were lower only in the CDA 2% groups. Water sorption was significantly increased at the 1.0% and 2.0% concentrations.

**Conclusions:**

We concluded that the incorporation of CDA into PMMA-based resins enabled the inhibition of *C. albicans* growth rate, did not alter the degrees of conversion of the tested resins and did not change the release of residual monomers.

## Introduction

A large variety of microorganisms take advantage of the environments generated by prostheses and use them as a substrate for colonization.^1^^,^^2^*Candida albicans,* one of these microorganisms, is an opportunistic pathogen capable of generating inflammatory responses in tissues, especially in immunocompromised patients.^3^^–^^6^ Furthermore, it is a fact that acrylic resins are porous and have low resistance to abrasion.^7^^,^^8^ Over time, increased surface roughness commonly leads to biofilm accumulation, creating a conducive environment to the development of microorganisms such as the *C. albicans*.^2^^,^^9^^,^^10^

The principal form to control and eliminate infections caused by *C. albicans* is the use of proper hygiene aids and drugs with antifungal activity. Among these drugs, chlorhexidine has proven antifungal action against *C. albicans* and the advantage of being effective even against strains that are resistant to other local antifungal agents available to treat this infection in the oral cavity.^3^^–^^5^^,^^11^^,^^12^

The chlorhexidine has been successfully used in Dentistry and is available in different formulations, including digluconate, hydrochloride, and diacetate formulations. The first formulation is most commonly used in mouthwashes^13^ because it is more soluble in water, while the last two formulations are more soluble in ethanol. The use of chlorhexidine is considered the gold standard antiseptic treatment for this agent has strong bactericidal and bacteriostatic capacities.^14^ Although the results of chlorhexidine use have proven satisfactory, this drug efficacy in the form of mouthwashes or gels depends directly on the patient's cooperation; thus, the expected result is not always obtained.^15^

For this reason, several examinations have investigated the potential use of drug delivery systems involving the incorporation of antifungal or antimicrobial agents into denture acrylic resins or soft liners.^16^^–^^18^ Silver nanoparticles, fluconazole,^16^ nystatin,^17^ ketoconazole^18^ and chlorhexidine^18^ are some substances that have been incorporated into polymers.

Chlorhexidine is one of the principal antifungals that has been tested in drug release systems following its incorporation into acrylic resins.^18^^–^^21^ Moreover, soft liners placed in dentures have been used as carriers for this drug in the treatment of denture stomatitis.^22^^,^^23^

However, few studies have tested the chlorhexidine diacetate (CDA) incorporation into auto-polymerized acrylic resins. Therefore, this study aims to assess this drug influence following its incorporation into auto-polymerized polymethyl methacrylate (PMMA)-based resins and the ability of such resins to release the drug by testing the following null hypotheses: a) chlorhexidine incorporation does not alter residual monomers leaching from the resins; b) chlorhexidine incorporated into rigid acrylic resins is not released from PMMA; c) chlorhexidine incorporation into acrylic resins does not enable the drug to inhibit *C. albicans* growth rate; d) chlorhexidine incorporation does not change the degrees of the conversion of the evaluated resins; and e) chlorhexidine incorporation does not alter the water sorption of PMMA-based acrylic resins.

## Methodology

### Test specimen preparation

To calculate sample size, SigmaPlot 14.0 tool with the test power set in 0.80 was used, and the values were obtained from the previous study by Bertollini, et al.^21^ (2014). According to the calculation, 3 samples were needed in each group for chlorhexidine release, residual monomer leaching, and antifungal activity; in addition, for the degree of conversion n=6 and water sorption n=10. In total, 200 samples were used.

**Figure 1:**
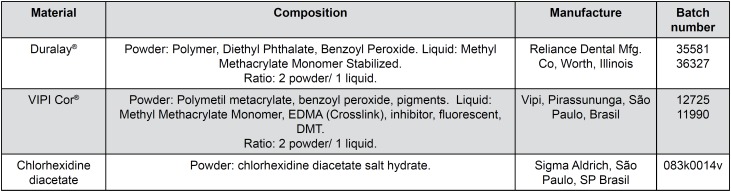
Materials used in this study, their composition, manufacturer and batch

The specimens were made with two self-polymerizable PMMA-based acrylic resins, i.e., Vipi Cor^®^ and DuraLay^®^, and comprised 8 groups involving the incorporation of 0.5%, 1.0% or 2.0% CDA salt into each resin and 2 control groups without the drug, as shown in Figure 1.

To make the test specimens, resins were manipulated according to the manufacturers’ recommendations in a ratio of 2 g polymer to 1 mL monomer. The chlorhexidine diacetate salt was separately weighed on a precision scale with 4-digit accuracy (Gehaka BG200; São Paulo, SP, Brazil) to obtain 0.5%, 1.0%, and 2.0% samples by weight for each polymer material. The powder/monomer/CDA proportions for each mixing group were as follows: 0.5% CDA, 5 g/2.5 mL/0.025 g; 1% CDA, 5 g/2.5 mL/0.05 g; and 2% CDA, 5 g/2.5 mL/0.1 g.

Initially, the chlorhexidine diacetate was dissolved in the monomer until achieving a homogeneous mixture. Subsequently, the polymer was added and mixed for 30 seconds.

### High-performance liquid chromatography (HPLC)

HPLC was used to measure the chlorhexidine release and residual monomer leaching from the acrylic resins, for all groups. For this reason, after manipulating the resins, they were placed in disc-shaped silicon molds measuring 10.0 mm in diameter and 3.0 mm in thickness. At the plate, three discs of each group were placed, and the samples were collected in triplicate.^21^^,^^24^^–^^26^ The specimens were polymerized for 5 min at ambient temperature.^24^

The specimens were stored individually in 24-well cell culture plates (TPP Techno Plastic Products; Trasadingen, Switzerland) containing 1 mL of sterile distilled water in each well. The volume of liquid used was sufficient to fully cover the surface of the resin disc. The storage liquid was maintained at 37°C and was changed every 48 hours to obtain a release curve.

Following, the assessment of chlorhexidine and residual monomer release, the solution was removed after 2 hours and measured via HPLC, associated with ultraviolet spectroscopy (UV). This procedure was repeated every 7 days to create a 28-day interval. The established time interval was based on chlorhexidine release in other release systems that are similar to the one tested here.^25^^,^^26^

The HPLC system consisted of a quaternary pump, degasser, automatic injector, column oven, and UV-DAD detector (Alliance HPLC system, Waters; Milford, MA, USA).

### Chlorhexidine release

The analysis proceeded through an SB C8 column (250x4.6 mm; 5 μm), and a monobasic sodium phosphate plug (0.03 M, pH 2.0, 80:20 v/v%) mobile phase was used at 2.0 mL/min flow rate. The chlorhexidine diacetate was detected at its maximum absorption, and the concentration was determined after constructing a calibration curve for the equipment with different drug concentrations used in this study.

### Residual monomer release

The analysis proceeded with a RP-18 column measuring 250 mm long by 2.5 mm in diameter with a mobile phase of 66% MeOH solution and 34% H_2_O at 2.0 mL/min flow rate. To quantify values, the pure monomer of each tested resin was used to construct a calibration curve that enabled the subsequent calculation of the concentrations as required by the ISO 1567 regulation. After performing the equipment calibration, monomers were detected at maximum absorption.

Once all the measurements had been obtained, data were plotted and statistically analyzed with the SPSS program (SPSS Inc.; Chicago, IL, USA). The differences between chlorhexidine and residual monomer releases into the storage solutions were determined by Kruskal-Wallis test followed by Dunn's *post-hoc* test for multiple comparisons. In all analyses, significance level was set at 5%.

### Antifungal activity

In total, 24 DuraLay^®^ and Vipi Cor^®^ disc-shaped samples were examined. The samples were divided into 4 groups for each materials with 0%, 0.5%, 1.0%, or 2.0% chlorhexidine, a total of 8 groups with 3 discs each.^21^

*Candida albicans* strain obtained from the Oswaldo Cruz Foundation (INCQS 40006, Lot 041240006) was used. Colonies were developed in brain heart infusion (BHI) cultures in aerobic incubation at 37°C. The agar diffusion test was performed using a technique previously described by Radnai, et al.^23^ (2010).

The agar was prepared and sterilized according to the manufacturer's standards. In a laminar flow hood, 25-30 mL of agar was transferred to 90x15-mm polystyrene sterile petri plates to obtain 4 mm of mean thickness. The plate covers were left slightly open to prevent the formation of moisture on their inner surfaces. After solidification of the media, plates were capped and placed in an oven at 37°C to ensure their sterility and then placed in a refrigerator until use. The agar diffusion test was performed using a technique previously described by Radnai, et al.^23^ (2010).

The surface received a prepared *C. albicans* suspension of 100 µl (10^8^-10^9^ CFU/mL) that was pipetted and inoculated by rubbing the swab across the sterile surface. Each group had three discs placed at the plate, and the samples were collected in triplicate. The specimens were distributed uniformly on the surface of each plate.^21^ Then, the plates with the samples were incubated at 37°C for 48 hours.

After this period, the inhibition zone diameters of the *C. albicans* growth generated around the resin discs were measured using a calibrated digital caliper (Mitutoyo; Tokyo, Japan) and reflected light.

In total, five measurements were performed for each disc, their diameter (10 mm) was then subtracted, and the mean of the 15 values for each concentration was calculated. The formation of an inhibition zone indicated the absence of *C. albicans* growth and thus demonstrated the inhibitory effect of the chlorhexidine incorporated into the PMMA-based resin.^21^

Data were entered into the SPSS program (SPSS Inc.; Chicago, IL, USA) to perform the statistical analyses. The differences between the *C. albicans* growth inhibition zones produced by the different concentrations of chlorhexidine were assessed after 48 hours using Kruskal-Wallis test followed by Dunn's *post-hoc* test for multiple comparisons. In all analyses, significance level was set at 5%.

### Degree of resin conversion

The degree of resin conversion was analyzed with Fourier transform infrared spectroscopy (FTIR) (Spectrum 100 Optica, PerkinElmer; Waltham, MA, USA) with an attenuated total reflectance element (ATR) coupled to a horizontal zinc selenide crystal (Pike Technologies; Madison, WI, USA) with a standard compression force of 100 N and 32 absorbance scans for each sample. Initially, an unpolymerized resin sample (no.=6)^26^^,^^27^ from each group was assessed by positioning it on the crystal and then performing 4 absorbance scans.

The obtained infrared spectra were assessed using an intensity of 1637 cm^−1^ and a peak at 2952 cm^−1^, which correspond to the double carbon bonds between the methacrylate and the simple connections made via polymerization, respectively^15^ as shown in Figure 1. Using the differences in the peak heights of the polymerized and unpolymerized samples and based on the fact that polymerization occurs from linked pairs breakdown and single bonds formation. The degree of conversion was calculated according to the following formula, and the values are reported as percentages (%):

$$\text{CD}\;(\%)\left(\frac{\frac{1.637{\text{cm}}^{-1}}{2.952\;{\text{cm}}^{-1}}\text{polymerized}}{\frac{1.637{\text{cm}}^{-1}}{2.952\;{\text{cm}}^{-1}}\text{unpolymerized}}\right)\times 100$$

The mean values for all groups were obtained and then subjected to a two-way ANOVA and subsequent Tukey tests with the predetermined α of 0.05.

### Water sorption

To measure the water sorption by the resins containing CDA, 10 disc-shaped specimens measuring 50 mm in diameter and 0.5 mm in thickness were prepared for each tested group. To obtain the specimens, the resins were manipulated in the same manner as those fabricated in the other tests. To achieve their working phases, the specimens were pressed in a muffle containing a stainless steel die press under an approximately 1250 kgf load and were deflasked after 1 hour.

After deflasking, an aluminum oxide-based grindstone driven by an electric motor (Talmax; Curitiba, PR, Brazil) was used to remove any roughness excess. At the end of this process, the specimen dimensions were measured with a digital caliper to ensure that they presented a maximum error below 0.5 mm in diameter and 0.05 mm in thickness.^28^

After obtaining the specimens, they were placed on a holder in a desiccator (Sigma Aldrich; St. Louis, MO, USA) at 37°C and weighed daily on an analytical balance (Thermo Fisher Scientific Co.; Waltham, MA, USA) with a precision of 0.0001 g until the weight was constant (m_0_). After this intrinsic moisture removal of resin was completed, each specimen was immersed in 100 mL of deionized water at a constant temperature of 37°C for 7 days, and the water was changed daily. After this period, the specimens were carefully blotted dry for one min to remove the water and weighed again to obtain the new resin weight (m_1_). The sorption calculation was then performed using the following mathematical formula:^29^

$$\text{WS}\left(\frac{\text{μg}}{{\underline{\text{mm}}}^{3}}\right)=\frac{m_{1}-m_{0}}{V_{0}}$$

m_0_ is the initial sample weight, and V_0_ is the initial sample volume.

The mean value of each group was obtained, and the results were then subjected to two-way ANOVA analysis with subsequent Tukey tests (p<0.05).

## Results

### High-performance liquid chromatography (HPLC)

**Figure 2:**
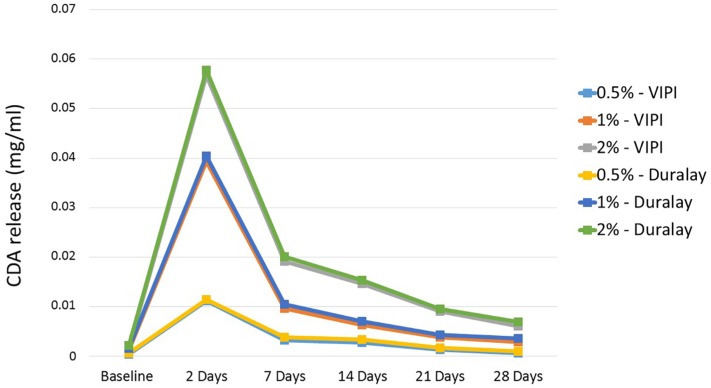
Profile of chlorhexidine diacetate release from acrylic resins

**Figure 3:**
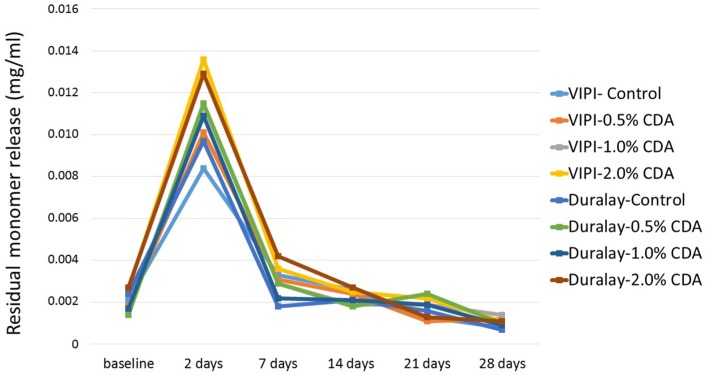
Profile of residual monomer release from resins containing chlorhexidine diacetate

The HPLC analysis results are presented in Figures 2 and 3. The profiles of the chlorhexidine diacetate release and residual monomer leaching from the acrylic resins containing chlorhexidine are also shown in the Graphs.

Shapiro-Wilk test revealed that two of the three reviewed factors (i.e., drug concentration and storage time) significantly changed chlorhexidine release rate (p<0.0001), but the material brand did not appear to significantly influence the release of the drug.

The residual monomer release results exhibited numerical changes; however, these differences were not statistically significant for any of the assessed drug concentrations (p>0.05).

### *C. albicans* inhibition zone - agar diffusion method

Based on the results shown in Table 1, both chlorhexidine diacetate resins were able to inhibit *C. albicans* growth rate because they induced an inhibition zone formation.

**Table 1:** Zone measurement in mm percentage of chlorhexidine diacetate resin

Resin	Percentage of chlorhexidinne			
	0%	0.5%	1.0%	2.0%
VIPI^®^	0^Aa^	1.240^Ab^	1.692^Abc^	1.972^Ac^
Duralay^®^	0^Aa^	1.839^Ab^	1.792^Abc^	2.240^Ac^

* Note: measurements followed by different letters (capital letters in the horizontal and lower case letters in the vertical directions differ among them; p<0.05)

Moreover, the statistical analyses revealed no significant differences between the two tested brands in terms of their abilities to inhibit *C. albicans*. Regarding the chlorhexidine concentration, all tested concentrations differed significantly from the control groups, which showed no inhibitory capacities. There were no significant differences between the 2% and 1% concentration groups or the 1% and 0.5% groups.

### Degree of conversion evaluation

**Figure 4:**
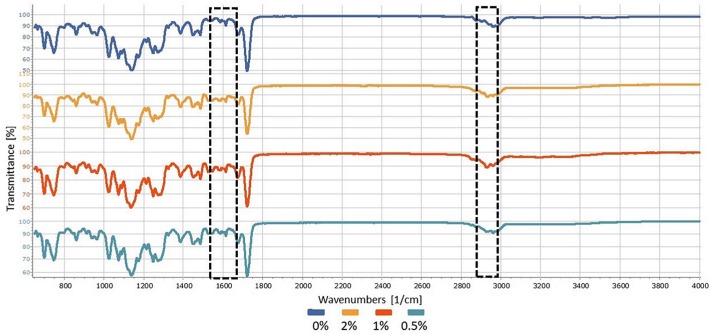
FTIR spectra of Duralay^®^ samples in different concentrations. The marked bands are associated with the double carbon bonds between the methacrylate (1,637 cm^−1^) and the simple connections made via polymerization (2,952 cm^−1^)

**Figure 5:**
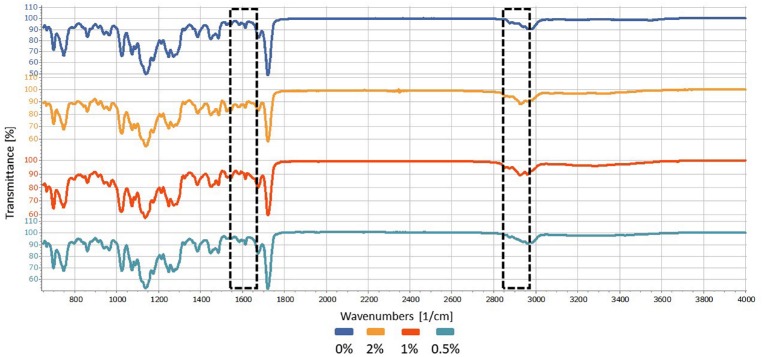
FTIR spectra of Vipi Cor^®^ samples in different concentrations. The marked bands are associated with the double carbon bonds between the methacrylate (1,637 cm^−1^) and the simple connections made via polymerization (2,952 cm^−1^)

Analyses of the degrees of the conversion of the acrylic resins indicated that the presence of the chlorhexidine salt caused no significant changes in resin conversion processes assessed in this study, which confirmed the initial hypothesis (p>0.05), as shown in Table 2, Figures 4 and 5.

**Table 2:** Degree of conversion of acrylic resins with chlorhexidine diacetate

Resin	Percentage of chlorhexidinne			
	0%	0.5%	1.0%	2.0%
VIPI^®^	91.5 ± 3.5^Aa^	90.8 ± 3.1^Aa^	90.2 ± 2.4^Aa^	88.6 ± 2.7^Aa^
Duralay^®^	92.2 ± 2.3^Aa^	92.1 ± 3.2^Aa^	91.8 ± 2.9^Aa^	89.4 ± 2.1^Aa^

* Note: measurements followed by different letters (capital letters in the horizontal and lower case letters in the vertical directions differ among them; p<0.05)

### Water sorption

The analyses of the sorption values of the resins (Table 3) revealed that the two resins behaved similarly when comparing samples with the same chlorhexidine concentrations. However, the 1% and 2% groups were significantly different from both the control and 0.5% group. Moreover, a significant difference was detected between the 1% and 2% groups.

**Table 3:** Sorption values of acrylic resins with chlorhexidine diacetate (ug/mm^3^)

Resin	Percentage of chlorhexidinne			
	0%	0.5%	1.0%	2.0%
VIPI^®^	18.6±2.7^Aa^	20.7±1.8^Aa^	23.5±2.9^Ba^	28.1±2.7^Ca^
Duralay^®^	19.2±3.2^Aa^	21.9±2.2^Aa^	24.1±3.1^Ba^	29.6±2.3^Ca^

* Note: measurements followed by different letters (capital letters in the horizontal and lower case letters in the vertical directions differ among them; p<0.05)

## Discussion

The results obtained, related to the drug release profiles, corroborated the kinetics previously described in the literature for the release of antifungal agents because the initial concentration of the drug generates a significantly higher diffusion gradient than it does during the remaining 28 days.^19^^,^^30^^–^^32^

The diffusion gradient changes the drug output due to the release mechanism of these compounds, which involves storage solution entry through the matrix chains, polymer network expansion, and drug dissolution, which is removed from the diffusion material.^33^

Conversely, the output of compounds from the internal portions might be hampered by the requirement that the drug transits through a large area of material. However, once dissolved, chlorhexidine particles leave pores in the polymer matrix, which enables diffusion from new to interconnected pores. This process promotes the contact of the acrylic surface with the medium, which allows maintaining the release level throughout the observed period.^33^

In contrast to the third null hypothesis, the results showed an antifungal effect of chlorhexidine diacetate incorporated into the tested materials that led to the inhibition of *C. albicans* growth rate in the BHI agar culture. No significant differences were observed between the inhibitory capacities of the different resins containing the same chlorhexidine concentrations. These findings were observed because drug and resins release capacities are very close, which is probably due to the similar compositions of the two assessed resins.

Furthermore, in two intra-group comparisons of CDA concentrations (i.e., 2% vs. 1% and 1% vs. 0.5%), no significant differences were established between the two resin brands.

Previous studies have demonstrated that the inhibition zone size tends to increase in proportion to the amount of antifungal agent incorporated into the material either due to direct contact of the specimen with the plate culture^16^^,^^34^^,^^35^ or with the storage solution into which the agar-containing wells are placed.^19^^,^^21^^,^^31^ However, in this study, although statistically different drug amounts were released by each group, no significant differences were found between the groups with concentrations of 2% vs. 1% or 1% vs. 0.5%.

It was postulated that chlorhexidine salt presence would not alter the kinetics of polymerization or the medium viscosity. These hypotheses were confirmed by the lack of significant alteration in either parameter. Furthermore, it was emphasized that no significant change in conversion between the materials would be observed, probably because of the similarity between the two resin compositions.^36^ However, a negative impact on the degree of conversion has been noted in studies that incorporated 10% chlorhexidine diacetate into resins; thus, it is likely that, at the concentrations used in this study, the amount of drug might have been insufficient to reduce radicals to a concentration that could change the final conversions of these materials.^36^^,^^37^

Although the evaluation of the degree of conversion with FTIR represents an important analysis, it should be noted that this test provides only an average measure of the double covalent bonds that are converted into saturated bonds and ignores the possible presence of double bonds in the residual monomer. Notably, in a portion of the monomer, the reaction may occur in only one of the double bonds and thereby reduce the final conversion without increasing leaching due to the connection with the polymer network. Although the influence of chlorhexidine incorporation on the degree of conversion of the resins was analyzed, assessment of the residual monomers release with HPLC will be important to establish this process correlation with subsequent assessment of cytotoxicity.^38^^,^^39^

Regarding the chlorhexidine salt influence on water sorption by the acrylic resins, those containing chlorhexidine exhibited higher water sorption rates than the control groups. This finding was emphasized by the statistically significant increases in the sorptions of the resins containing 2.0% chlorhexidine diacetate compared with those containing 1.0%. These changes confirmed the results of previous studies^32^ reporting that as the incorporation of chlorhexidine salt into these resins increases, the sorption process also increases. This increase is supported by the fact that the salt loss leaves micrometric pores in the material structure increasing the surface of contact area with the environment, which enhances the water intake process into the material.^32^

The sorption rates of both resins showed similarity between the groups with the same concentrations of chlorhexidine. This finding probably resulted from the similar conversions and compositions of the resins used. The homogeneity between the resin sorption rates probably was caused by the similar kinetics release, since the main output control of the drug from the matrix was mediated by the entry of water, which enabled the drug dissolution and caused its subsequent output by the diffusion process, generated by the concentration gradient. This process caused matrix swelling when water sorption by the material occurred.^32^ Therefore, sorption may facilitate drug release into the aqueous environment into which it is incorporated.

Another influential factor in the sorption process is the degree of conversion. Such conversion has previously been elucidated in the literature, and the findings indicate that poorly converted polymers exhibit reduced mechanical properties and suffer greater swelling and solvent sorption.^37^^,^^40^ However, for similar conversions between groups, the increased sorption process occurred specifically due to the formation of pores rather than the drug output.

Based on the foregoing discussion, it can be established that the similar release profiles/kinetics of the DuraLay^®^ (PEMA/PMMA) and VIPI Cor^®^ (PMMA) materials occurred because the two resins exhibit very similar rates of water sorption, compositions, and conversion rates, which probably allowed for a swelling rate similar to those of their matrices.

In this study, the sustained release of chlorhexidine from the PMMA materials suggests chlorhexidine may be convenient for reducing biofilm development on the material surfaces while not affecting their physical properties. Moreover, chlorhexidine could be used in dentures and temporary restorative materials, which would remove the dependence on patient's compliance.

One limitation of this study is that the *in vitro* results should be carefully extrapolated to the clinical setting considering the following restrictions. First, immersion in distilled water or other solvents does not reproduce the clinically observed magnitudes or changing rates in material properties. Second, the loss of compounds that may leach occurs faster *in vivo* due to the oral environment as well as the eating and hygiene habits of the patient. Consequently, only future *in vivo* clinical studies can determine the performances of modified resilient materials.

## Conclusion

Based on the methodology used and results obtained in this study, we conclude the following: a) the incorporation of chlorhexidine into PMMA-based resins allows for its subsequent release from the matrices, which enables the resins to inhibit *C. albicans* growth rate; b) the incorporation of chlorhexidine did not change the degrees of conversion of the resins and therefore did not change their residual monomer release values; and c) drug incorporation changed the sorption values of PMMA-based acrylic resins.

## References

[1] 1- Gendreau L, Loewy ZG. Epidemiology and etiology of denture stomatitis. J Prosthodont. 2011;20(4):251-60. 10.1111/j.1532-849X.2011.00698.x 21463383

[2] 2-Carlsson GE, Omar R. The future of complete dentures in oral rehabilitation. A critical review. J Oral Rehabil. 2010;37(2):143-56. 10.1111/j.1365-2842.2009.02039.x 20002536

[3] 3- Coco BJ, Bagg J, Cross LJ, Jose A, Cross J, Ramage G. Mixed *Candida albicans* and *Candida glabrata* populations associated with the pathogenesis of denture stomatitis. Oral Microbiol Immunol. 2008;23(5):377-83. 10.1111/j.1399-302X.2008.00439.x 18793360

[4] 4- Gleiznys A, Zdanavičiene E, Žilinskas J. *Candida albicans* importance to denture wearers. A literature review. Stomatologija. 2015;17(2):54-66. 26879270

[5] 5- Redding S, Bhatt B, Rawls HR, Siegel G, Scott K, Lopez-Ribot J. Inhibition of *Candida albicans* biofilm formation on denture material. Oral Surg Oral Med Oral Pathol Oral Radiol Endod. 2009;107(5):669-72. 10.1016/j.tripleo.2009.01.021 19426921

[6] 6-Aoun G, Cassia A, Berberi A. Effectiveness of a chlorhexidine digluconate 0.12% and cetylpyridinium chloride 0.05% solution in eliminating *Candida albicans* colonizing dentures: a randomized clinical *in vivo* study. J Contemp Dent Pract. 2015;16(6):433-6. 10.5005/jp-journals-10024-1702 26323444

[7] 7- Arikan A, Ozkan YK, Arda T, Akalin B. Effect of 180 days of water storage on the transverse strength of acetal resin denture base material. J Prosthodont. 2010;19(1):47-51. 10.1111/j.1532-849X.2009.00495.x 20002973

[8] 8- Cevik P, Yildirim-Bicer AZ. The effect of silica and prepolymer nanoparticles on the mechanical properties of denture base acrylic resin. J Prosthodont. 2018;27(8):763-70. 10.1111/jopr.12573 27898997

[9] 9- Figueiral MH, Azul A, Pinto E, Fonseca PA, Branco FM, Scully C. Denture-related stomatitis: identification of a etiological and predisposing factors - a large cohort. J Oral Rehabil. 2007;34(6):448-55. 10.1111/j.1365-2842.2007.01709.x 17518980

[10] 10- Nett JE, Marchillo K, Spiegel CA, Andes DR. Development and validation of an *in vivo Candida albicans* biofilm denture model. Infect Immun. 2010;78(9):3650-9. 10.1128/IAI.00480-10 PMC2937450 20605982

[11] 11- Mima EG, Pavarina AC, Vargas FS, Giampaolo ET, Machado AL, Vergani CE. Effectiveness of chlorhexidine on the disinfection of complete dentures colonised with fluconazole-resistant *Candida albicans: in vitro* study. Mycoses. 2011;54(5):506-12. 10.1111/j.1439-0507.2010.01968.x 21605178

[12] 12- Salim N, Moore C, Silikas N, Satterthwaite J, Rautemaa R. Chlorhexidine is a highly effective topical broad-spectrum agent against *Candida* spp. Int J Antimicrob Agents. 2012;41(1):65-9. 10.1016/j.ijantimicag.2012.08.014 23084595

[13] 13- Eick S, Goltz S, Nietzsche S, Jentsch H, Pfister W. Efficacy of chlorhexidine digluconate-containing formulations and other mouthrinses against periodontopathogenic microorganisms. Quintessence Int. 2011;42(8):687-700. 21842009

[14] 14- Ryalat S, Darwish R, Amin W. New form of administering chlorhexidine for treatment of denture-induced stomatitis. Ther Clin Risk Manag. 2011;7:219-25. 10.2147/TCRM.S18297 PMC3132092 21753884

[15] 15- Souza RF, Regis RR, Nascimento C, Paranhos HF, Silva CH. Domestic use of a disclosing solution for denture hygiene: a randomised trial. Gerodontology. 2010;27(3):193-8. 10.1111/j.1741-2358.2009.00309.x 19545320

[16] 16- Salim N, Moore C, Silikas N, Satterthwaite J, Rautemaa R. Candidacidal effect of fluconazole and chlorhexidine released from acrylic polymer. J Antimicrob Chemother. 2013;68(3):587-92. 10.1093/jac/dks452 23171950

[17] 17- Al-Haddad, R. Vahid Roudsari R, Satterthwaite JD. Fracture toughness of heat cured denture base acrylic resin modified with chlorhexidine and fluconazole as bioactive compounds. J Dent. 2014;42(2):180-4. 10.1016/j.jdent.2013.11.007 24269832

[18] 18- Bueno MG, Urban VM, Barbério GS, Silva WJ, Porto VC, Pinto L, et al. Effect of antimicrobial agents incorporated into resilient denture relines on the *Candida albicans* biofilm. Oral Dis. 2015;21(1):57-65. 10.1111/odi.12207 24219354

[19] 19- Amin WM, Al-Ali MH, Salim NA, Al-Tarawneh SK. A new form of intraoral delivery of antifungal drugs for the treatment of denture-induced oral candidosis. Eur J Dent. 2009;3(4):257-66. PMC2761155 19826596

[20] 20- Hotta J, Garlet GP, Cestari TM, Lima JF, Porto VC, Urban VM, et al. *In vivo* biocompatibility of an interim denture resilient liner containing antifungal drugs. J Prosthet Dent. 2019;121(1):135-42. 10.1016/j.prosdent.2018.02.005 30646999

[21] 21- Bertolini MM, Portela MB, Curvelo JA, Soares RM, Lourenço EJ, Telles DM. Resins-based denture soft lining materials modified by chlorhexidine salt incorporation: an *in vitro* analysis of antifungal activity, drug release and hardness. Dent Mater. 2014;30(8):793-8. 10.1016/j.dental.2014.05.004 24933229

[22] 22- Salim N, Moore C, Silikas N, Satterthwaite JD, Rautemaa R. Fungicidal amounts of antifungals are released from impregnated denture lining material for up to 28 days. J Dent. 2012;40(6):506-12. 10.1016/j.jdent.2012.02.016 22390981

[23] 23- Radnai M, Whiley R, Friel T, Wright PS. Effect of antifungal gels incorporated into a tissue conditioning material on the growth of *Candida albicans*. Gerodontology. 2010;27(4):292-6. 10.1111/j.1741-2358.2009.00337.x 19732159

[24] 24-Tallury P, Alimohammadi N, Kalachandra S. Poly(ethylene-co-vinyl acetate) copolymer matrix for delivery of chlorhexidine and acyclovir drugs for use in the oral environment: effect of drug combination, copolymer composition and coating on the drug release rate. Dent Mater. 2007;23(4):404-9. 10.1016/j.dental.2006.02.011 16556460

[25] 25- Kalachandra S, Lin DM, Stejskal EO, Prakki A, Offenbacher S. Drug release from cast films of ethylene vinyl acetate (EVA) copolymer: stability of drugs by 1H NMR and solid state 13C CP/MAS NMR. J Mater Sci Mater Med. 2005;16(7):597-605. 10.1007/s10856-005-2529-1 15965590

[26] 26- Li J, Barrow D, Howell H, Kalachandra S. *In vitro* drug release study of methacrylate polymer blend system: effect of polymer blend composition, drug loading and solubilizing surfactants on drug release. J Mater Sci Mater Med. 2010;21(2):583-8. 10.1007/s10856-009-3899-6 19856082

[27] 27- Bural C, Aktaş E, Deniz G, Ünlünçerçi Y, Kizilcan N, Bayraktar G. Effect of post-polymerization heat treatments on degree of conversion, leaching residual MMA and *in vitro* cytotoxicity of autopolymerizing acrylic repair resin. Dent Mater. 2011;27(11):1135-43. 10.1016/j.dental.2011.08.007 21920593

[28] 28- International Standard Organization. ISO 1567:1999. Dentistry-denture base polymers. Geneva: International Organization for Standardization; 1999.

[29] 29- Barszczewska-Rybarek I, Chladek G. Studies on the curing efficiency and mechanical properties of Bis-GMA and TEGDMA nanocomposites containing silver nanoparticles. Int J Mol Sci. 2018;19(12). pii: E3937. 10.3390/ijms19123937 PMC6320904 30544584

[30] 30- Villar CC, Lin AL, Cao Z, Zhao XR, Wu LA, Chen S, et al. Anticandidal activity and biocompatibility of a rechargeable antifungal denture material. Oral Dis. 2013;19(3):287-95. 10.1111/odi.12000 PMC3654054 22957799

[31] 31- Darwish RM, Amin WM, Al-Ali MH, Salem NA. Study of the elution of fluconazole from a self-polymerizing acrylic resin and its activity against resistant *Candida albicans*. J Mater Sci Mater Med. 2011;22(8):1885-90. 10.1007/s10856-009-3893-z 19844777

[32] 32- Patel MP, Cruchley AT, Coleman DC, Swai H, Braden M, Williams DM. A polymeric system for the intra-oral delivery of an anti-fungal agent. Biomaterials. 2001;22(8):2319-24. 10.1016/s0142-9612(00)00367-7 11511028

[33] 33- Tallury P, Airrabeelli R, Li J, Paquette D, Kalachandra S. Release of antimicrobial and antiviral drugs from methacrylate copolymer system: effect of copolymer molecular weight and drug loading on drug release. Dent Mater. 2008;24(2):274-80. 10.1016/j.dental.2007.05.008 17628658

[34] 34- Arnold RR, Wei HH, Simmons E, Tallury P, Barrow DA, Kalachandra S. Antimicrobial activity and local release characteristics of chlorhexidine diacetate loaded within the dental copolymer matrix, ethylene vinyl acetate. J Biomed Mater Res B Appl Biomater. 2008;86(2):506-13. 10.1002/jbm.b.31049 18335433

[35] 35- Salim N, Silikas N, Satterthwaite JD, Moore C, Ramage G, Rautemaa R. Chlorhexidine-impregnated PEM/THFM polymer exhibits superior activity to fluconazole-impregnated polymer against *Candida albicans* biofilm formation. Int J Antimicrob Agents. 2013;41(2):193-6. 10.1016/j.ijantimicag.2012.09.006 23127479

[36] 36- Riggs PD, Braden M, Patel M. Chlorhexidine release from room temperature polymerizing methacrylate systems. Biomaterials. 2000;21(4):345-51. 10.1016/s0142-9612(99)00187-8 10656315

[37] 37- Salim N, Satterthwaite J, Rautemaa R, Silikas N. Impregnation with antimicrobials has an impact on degree of conversion and colour stability of acrylic liner. Dent Mater J. 2012;31(6):1008-13. 10.4012/dmj.2012-121 23207208

[38] 38- Goiato MC, Freitas E, Santos D, Medeiros R, Sonego M. Acrylic resin cytotoxicity for denture base - literature review. Adv Clin Exp Med. 2015;24(4):679-86. 10.17219/acem/33009 26469114

[39] 39- Bural C, Aktaş E, Deniz G, Ünlüçerçi Y, Bayraktar G. Effect of leaching residual methyl methacrylate concentrations on *in vitro* cytotoxicity of heat polymerized denture base acrylic resin processed with different polymerization cycles. J Appl Oral Sci. 2011;19(4):306-12. 10.1590/S1678-77572011005000002 PMC4223779 21956586

[40] 40- Bayraktar G, Guvener B, Bural C, Uresin Y. Influence of polymerization method, curing process, and length of time of storage in water on the residual methyl methacrylate content in dental acrylic resins. J Biomed Mater Res B Appl Biomater. 2006;76(2):340-5. 10.1002/jbm.b.30377 16161124

